# Deep Brain Stimulation for Post-Hypoxic Myoclonus: A Case Correlating Local Field Potentials to Clinical Outcome

**DOI:** 10.5334/tohm.999

**Published:** 2025-04-24

**Authors:** Harleen Kaur, Tim J. Goble, Albert Fenoy, Ritesh A. Ramdhani

**Affiliations:** 1 Department of Neurology, Louisiana State University Health Shreveport, Shreveport, LA, USA; 2 Medtronic Neuromodulation, Medtronic, Minneapolis, MN, USA; 3 Department of Neurological Surgery, Donald and Barbara Zucker School of Medicine at Hofstra/Northwell, Manhasset, NY, USA; 4 Department of Neurology, Donald and Barbara Zucker School of Medicine at Hofstra/Northwell, Manhasset, NY, USA

**Keywords:** post-hypoxic myoclonus, deep brain stimulation, local field potentials

## Abstract

**Background:**

Post-hypoxic myoclonus (PHM) is characterized by generalized myoclonus after hypoxic brain injury. PHM is often functionally impairing and refractory to medical therapies. There are a handful of reports utilizing deep brain stimulation (DBS) to treat medically refractory PHM.

**Case Report:**

A 56-year-old woman developed PHM following an anoxic brain injury. Utilizing a stimulating and sensing DBS system, we show clinical improvement in myoclonus at 6 months and correlate it to local field potential (LFP) activity.

**Discussion:**

We present the first case to utilize DBS sensing to correlate LFP activity to myoclonus improvement. Our case contributes to the growing evidence of DBS for PHM.

## Introduction

Post hypoxic myoclonus (PHM) or Lance Adams Syndrome is a critical neurological sequelae of brain injury following cardiac arrest, which impacts the long term care and neurological outcome in these patients [1]. First described in 1963 by Lance and Adams, PHM is defined as sudden onset of focal or generalized myoclonus present at rest, action or provoked by stimulation following hypoxic brain injury secondary to cardiopulmonary arrest [2,3]. While myoclonic status epilepticus tends to emerge acutely and have a poor prognosis, subacute onset of post hypoxic myoclonus is often seen within days and weeks following brain injury and can persist chronically [4].

The approach to the treatment of PHM has been primarily with antiepileptic medications [5,6,7,8]. However, there have been several reports of utilizing off-label use of deep brain stimulation (DBS) [9,10]; a well-established treatment for both hypo- and hyperkinetic movement disorders, including tremor, Parkinson’s disease, dystonia and epilepsy [11,12,13,14]. We report the first case correlating a patient’s improvement to their physiology, as measured by in newer embedded sensing DBS systems. Our patient showed improvement in her rest myoclonus following DBS and best medical management, which correlated with reduced local field potential activity. This report provides further evidence to the growing cases of DBS treating PHM, as well as insights into the chronic physiology of PHM disorder.

### Case Description

This is a 56-year-old female who suffered anoxic brain injury secondary to suicide attempt. She was resuscitated and treated in an intensive care unit for 11 days. She underwent successful extubation and did not require percutaneous gastrostomy. However, her mobility was markedly limited, and she was wheelchair bound. Myoclonus emerged several weeks following discharge from the hospital. Electroencephalogram (EEG) showed frequent myoclonic jerks without ictal correlate and generalized epileptiform discharges that are occasionally associated with myoclonic jerks. Brain magnetic resonance imaging demonstrated cortical atrophy with normal diffusion weighted imaging. The patient was seen in our center two years following the anoxic event. On examination (refer to Video 1), there were generalized myoclonic jerks at rest in her arms and legs with severe action myoclonus when reaching for objects or holding a cup. There was tactile induced myoclonus elicited in the upper limbs. Her speech consisted of frequent breaks and inspiratory gasps along with truncal jerks suggested of diaphragmatic myoclonus. There was orthostatic myoclonus in her legs when she stood, and her gait was ataxic and unsteady. Her myoclonic drug regimen included clonazepam 2.5 mg daily, levetiracetam 1000 mg daily, zonisamide 300 mg daily, and valproate; the latter was discontinued because of lack of tolerance.

**Video 1:** **The patient at pre-DBS**. There is multifocal myoclonus in the limbs and trunk at rest and with action. Asterixis and orthostatic myoclonus are also present.

Due to the refractory nature of her condition, preservation of her cognition, and poor quality of life, a decision was made—in counsel with the patient—to pursue deep brain stimulation of globus pallidus interna (GPi). This target was selected based on the team’s experience and the published evidence in the literature of treating myoclonus with GPi-DBS.

### Surgery and DBS Programming

The patient was implanted under anesthesia with bilateral GPi-DBS with SenSight™ leads and Percept™ PC implantable pulse generator IPG (Medtronic, Minneapolis, MN) two years after the anoxic event. The DBS IPG provides local field potential (LFP) sensing capabilities and offers an opportunity to understand patient and disease pathology while providing stimulation.

Chronic recording was initiated post DBS implantation at 10 Hz to allow the clinician to observe changes in LFP activity until the initial programming four weeks later. At the first programming visit, the LFP survey was conducted bilaterally for each available DBS contact. The highest observed LFP activity was used, and confirmed with anatomical imaging, to determine initial contact selection (contact 2 and 9). Initial parameters consisted of a pulse width of 90 µs and frequency of 130 Hz. Stimulation was tolerated above a threshold amplitude of 3.5 mA for both contacts 2 and 9.

### LFP Recording Paradigm

A recording paradigm was designed for the initial and all subsequent visits, which included reviewing chronic 10-min LFP averages at the highest spectral power (11 Hz) bilaterally, and a ~1 min LFP streaming of raw µVolt LFP signal, sampled at 250 Hz. LFP streaming was also performed during all subsequent changes to programming parameters during each clinic visit. Based on the notion that PHM has cortical and subcortical pathology, 11 Hz (alpha, subcortical) and 50 Hz (gamma, cortical) was chosen as a proxy to evaluate changes in broadband activity between clinic visits. At each visit, the patient was physically evaluated and her examination recorded. Blinded Unified Myoclonus Rating Scale (UMRS) was conducted on the video recordings to evaluate myoclonus at rest, with action, as well as global disability and negative myoclonus severity scores.

### Longitudinal LFP and UMRS Assessment and Analysis

The patient had four neurological visits within the first 6-months of DBS treatment: initial programming (three weeks after IPG and electrode implantation) followed by 1, 3, and 6-month follow-ups. Video 2 demonstrates the 6-month post-surgical examination with the following DBS stimulation parameters: Contact 2–C+: amplitude 3.5 mA, Pulse width (PW) 90 µs, Frequency 130 Hz; Contact 9– C+: amplitude 3.5 mA, Pulse width (PW) 90 µs, Frequency 130 Hz.

**Video 2:** **6-month post GPI-DBS**. Marked reduction in rest and action myoclonus in the trunk and limbs. Gait improves with less assistance needed.

**Figure 1:**
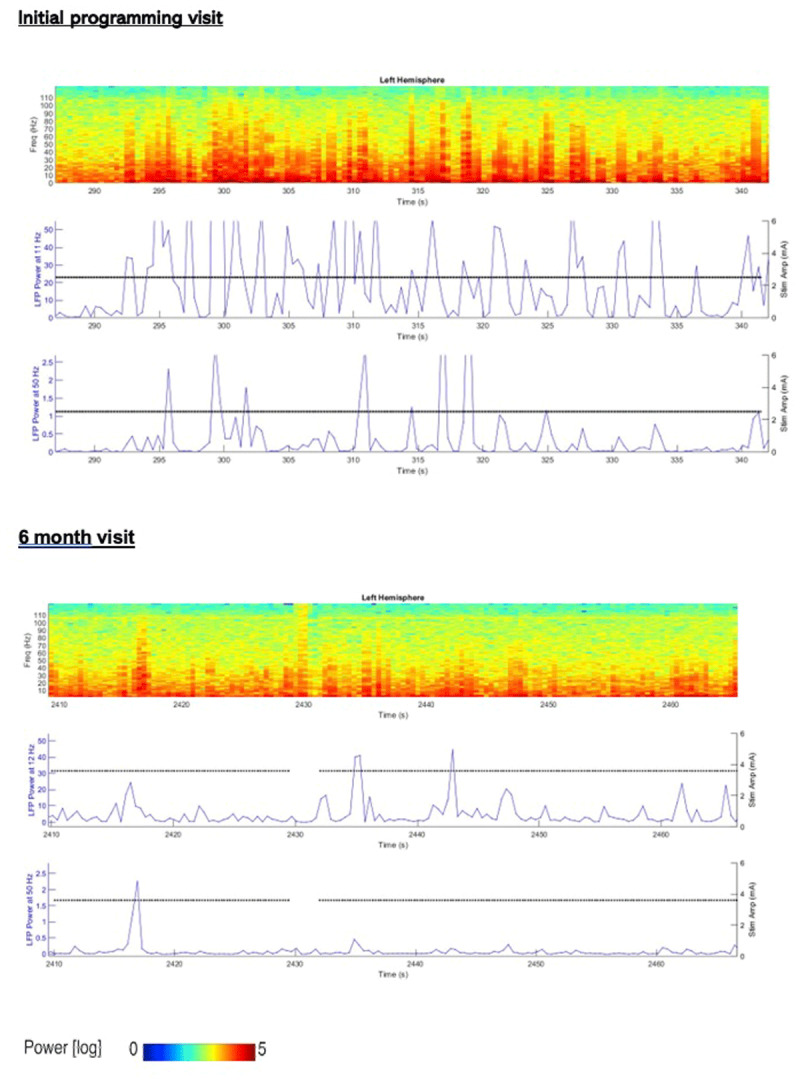
55sec of LFP spectrogram (y-axis represents frequency, 0–125 Hz and the color gradient is scaled from low power (Blue) to high LFP power (Red) normalized signal for a given time and frequency) and LFP power (LSB (analog to digital converter output)) from Left Gpi-DBS sensing electrode at 11–12 Hz and 50 Hz (top to bottom). The black solid line indicates stimulation current.

**Figure 2:**
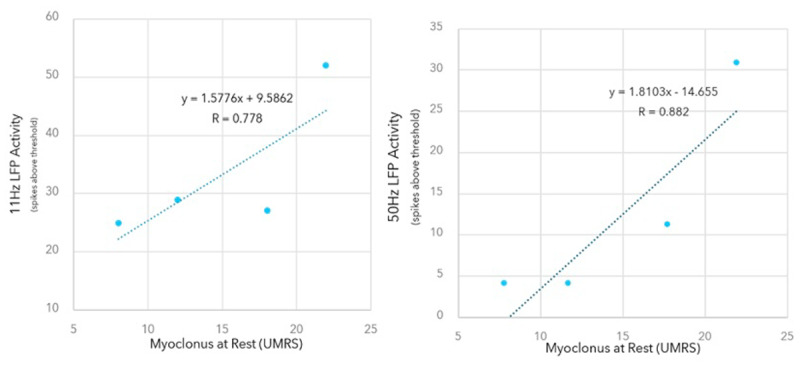
Correlation of rest and action myoclonus component of UMRS with LFP activity at 11 Hz and 50 Hz.

Streaming from programming sessions show 55 seconds of raw µVolt LFP power, sampled at 250 Hz, LFP spectrogram (0–125 Hz) and LFP power at 11 Hz and 50 Hz (± 2.5 Hz) sampled at 2 Hz; initial programming visit and at the 6-month visit (Figure 1). Unified Myoclonus Rating Scale (UMRS) were recorded for each visit (Table 1). Around 2-months post DBS stimulation, the patient’s nursing home changed her medication without consultation with the movement disorder neurologist and therefore, the patient presented with worsening myoclonus for the 3-month visit. However, the worsened UMRS scores on this visit correlated with increased LFP activity at both 11 Hz and 50 Hz. A Pearson correlation was performed on the rest and action components of the UMRS with LFP activity at 11- and 50-Hz (Figure 2). Rest myoclonus was highly correlated to LFP activity at 11 Hz and 50 Hz (Pearson r = 0.78 and 0.881), respectively. At 6 months follow-up, the patient showed 33% improvement in rest myoclonus, 66% improvement in negative myoclonus, and 50% improvement in the global disability score based on the UMRS.

**Table 1:** UMRS from each clinic visit.


	MYOCLONUS AT REST	MYOCLONUS WITH ACTION							GLOBAL DISABILITY	NEGATIVE MYOCLONUS SCORE	NEGATIVE MYOCLONUS SEVERITY

		R ARM	L ARM	R LEG	L LEG	ARISING	STANDING	WALKING			

Initial Programming	18	16	16	16	12	16	12	12	4	1	3

1-month follow-up	8	9	4	1	0	—	—	6	2	0	1

3-month follow-up	22	9	6	—	—	—	6	12	2	1	2

6-month follow-up	12	9	4	9	6	—	4	9	2	0	1

%change: Baseline to 6-month	33	44	75	44	50	—	67	25	50	100	66


## Discussion

This is the first known PHM case study to reveal a high correlation of rest myoclonus reduction with LFP suppression activity at both alpha and gamma frequency peaks using bilateral GPi-DBS.

While electrophysiological studies were not conducted, the phenomenology of the case, specifically the presence of action induced multifocal myoclonus, orthostatic myoclonus, and stimulus sensitive myoclonus suggested a cortical origin; the presence of rest myoclonus pointed to subcortical generators. The epileptiform discharges that were variably associated with myoclonic jerks on EEG further support the presence of cortical excitability to which an excitatory-inhibitory imbalance of the primary motor cortex (M1) has been shown to underlie cortical myoclonus [15].

Brown and Marsden [16] presented evidence that cortical activity in repetitive myoclonus is rhythmic and can drive a concomitant train of rhythmic EMG burst activity with a frequency as high as 50 Hz. Arrhythmic positive and negative myoclonus are [17] associated with enhanced motor cortex activity (16–20 Hz) as well. Uozumi et al. [18] reported a cortical hyper oscillatory pattern of approximately 50 Hz in a post-hypoxic myoclonic individual. Rhythmic oscillatory activation of cortical neurons (ranging 20–50 hz) projecting to the pyramidal tracts [19,20] exist in primates and healthy humans and are tied to muscle contractions and voluntary movement. Therefore, this EMG-EEG coherence evidence suggests that the nascent oscillatory activity between the brain and muscle is exaggerated in the diseased or injured state.

Subcortical dysfunction in PHM is less well characterized and physiologically delineated. It has been implicated in PHM through fluorodeoxyglucose positron emission tomography findings of elevated glucose metabolism in the bilateral ventrolateral thalami and pons [21] as well as histologically confirmed in the rat arrest model, whereby degeneration in both pyramidal cells of layers III and IV of the cerebral cortex and reticular thalamus along with extensive Purkinje cell damage in the cerebellum [22] were seen. While oscillatory behavior of a subcortical generator in PHM and the impact of cerebellar dysfunction has on cortical rhythms are unclear, the oscillatory correlations reported in this case suggests a possible pallidal – cortical coupling that may be informing the cortical-muscular coherence. Even though pyramidal tract origins of descending motor activity in myoclonus have been shown, we cannot exclude the possibility that these findings may support the role of cortico-reticulospinal pathways in influencing myoclonus generation and stimulation response. Additional electrophysiological studies are not only necessary to expand understanding of these subcortical-cortical circuits in PHM, but will enable advancement of existing treatments such as DBS.
